# Onsite versus home-office: differences in sleep patterns according to workplace

**DOI:** 10.1007/s11818-023-00408-5

**Published:** 2023-06-06

**Authors:** Naomi Staller, Mirja Quante, Helen Deutsch, Christoph Randler

**Affiliations:** 1 Department of Biology, University of Tuebingen, Tuebingen, Germany; 2 Department of Neonatology, University of Tuebingen, Tuebingen, Germany

**Keywords:** Actigraphy, Coronavirus disease 2019 pandemic, Sleep hygiene, Remote working, Circadian rhythm, Aktigraphie, COVID-19 Pandemie, Schlafhygiene, Remote-Arbeit, Zirkadianer Rhythmus

## Abstract

**Background and objective:**

This study aimed to evaluate the sleep patterns of students and employees working onsite versus those working from home during the COVID-19 pandemic using actigraphy.

**Methods:**

A total of 75 students/employees (onsite: *N* = 40, home-office: *N* = 35; age range: 19–56 years; 32% male; 42.7% students, 49.3% employees) were studied between December 2020 and January 2022 using actigraphy, a sleep diary, and an online questionnaire assessing sociodemographics and morningness–eveningness. Independent-sample *t*-tests, paired-sample tests, and a multivariate general linear model adjusting for age (fixed factors: sex and work environment) were applied.

**Results:**

Overall, onsite workers had significantly earlier rise times (7:05 [SD: 1:11] versus 7:44 [1:08] hours) and midpoints of sleep (2:57 [0:58] versus 3:33 [0:58] hours) on weekdays compared to home-office workers. Sleep efficiency, sleep duration, variability of sleep timing, and social jetlag did not differ between the groups.

**Discussion:**

Home-office workers showed a delay in sleep timing that did not affect any other sleep parameters such as sleep efficiency or nighttime sleep duration. The work environment had only marginal impact on sleep patterns and thus sleep health in this sample. Sleep timing variability did not differ between groups.

**Supplementary Information:**

The online version of this article (10.1007/s11818-023-00408-5) contains supplementary material 1 and 2, which is available to authorized users.

## Abstract (de)

**Hintergrund und Ziel:**

In dieser Studie wurde das Schlafverhalten von Studierenden und Angestellten, die während der COVID-19-Pandemie vor Ort oder von zu Hause aus arbeiteten, aktigraphisch untersucht.

**Methoden:**

Untersucht wurden 75 Studierende/Angestellte (vor Ort: *N* = 40, Home-Office: *N* = 35, Alter: 19–56 Jahre, 32 % männlich, 42,7 % Studierende, 49,3 % Angestellte) zwischen Dezember 2020 und Januar 2022 mittels Aktigraphie, Schlaftagebuch und Online-Fragebogen zur Erfassung von soziodemographischen Daten und Morningness-Eveningness. Verwendet wurden *t*‑Tests für unabhängige Stichproben, Tests für gepaarte Stichproben sowie ein altersbereinigtes multivariates allgemeines lineares Modell (feste Faktoren: Geschlecht und Arbeitsumgebung).

**Ergebnisse:**

Insgesamt standen die Vor-Ort-Arbeitenden an Wochentagen signifikant früher auf (7:05 [SD 1:11] vs. 7:44 [1:08] Stunden) und hatten einen signifikant früheren Schlafmittelpunkt (2:57 [0:58] vs. 3:33 [0:58] Stunden) als die im Home-Office-Arbeitenden. Schlafeffizienz, Schlafdauer, Schlafvariabilität und sozialer Jetlag unterschieden sich nicht zwischen den Gruppen.

**Diskussion:**

Im Home-Office arbeitende Teilnehmende zeigten eine Verzögerung des Schlafzeitpunkts, die sich nicht auf andere Schlafparameter, wie Schlafeffizienz oder nächtliche Schlafdauer, auswirkte. Die Arbeitsumgebung hatte in dieser Stichprobe nur einen geringen Einfluss auf das Schlafverhalten und damit auf die Schlafgesundheit. Die Variabilität der Schlafzeiten unterschied sich nicht zwischen den Gruppen.

Sleep is critical for regular functioning of physical and mental health in humans [1]. It is important for maintaining long-term health through physical/cerebral functioning (sleep deprivation causes, e.g., an increased prevalence of noncommunicable diseases such as obesity [2] or cardiovascular problems [3]), and short term health by, e.g., minimizing the risk of traffic accidents related to drowsiness [4, 5].

With the onset of the coronavirus disease 2019 (COVID-19) pandemic, mental stress increased enormously due to fears about the future, about relatives, or about one’s own health. Since stress and anxiety have a strong negative impact on sleep, some research groups have reported a deterioration in sleep health (see [6] for a review). In addition, increased workloads in some professions (e.g., medical staff) have also taken their toll, leading to more sleep problems and poorer sleep health [6].

The pandemic changed the professional situation of students and employees. Remote working (hereinafter also referred to as the situation of students studying at home) as well as the closure of many business sectors (e.g., body-related services) affected everyday life, including sleep. It was shown that the shift to remote working and the associated later work start allowed participants to adjust their sleep times to their own circadian rhythm. As a result, students and employees were able to synchronize their biological and social clocks, thereby improving sleep health (e.g., [7–15]). In addition to the positive effects of synchronizing social and biological timing, on-demand teaching has been shown to lead to a loss of sleep timing regularity [14], which, in turn, can have negative effects on sleep and overall health (e.g., [16, 17]). In contrast, Ramírez-Contreras and colleagues [18] reported that the hybrid system of virtual and face-to-face activity was beneficial for students in terms of sleep regularity and eating habits, resulting in longer sleep duration on weekdays and reductions in sleep deprivation. Furthermore, increased screen time due to remote working may also have disrupted and delayed sleep onset due to more blue light exposure (e.g., [19, 20]) and less outdoor light exposure [21]. However, increased time spent outdoors in home-office workers has also been reported [22]. So far, there are few data on the effect of the working environment on sleep patterns during the COVID-19 pandemic in Germany.

The present study builds on the preliminary data and literature available and compares the sleep patterns of participants in two different working environments. We hypothesized later/more irregular sleep timing and midpoints of sleep, longer nighttime sleep duration, worse sleep efficiency, and less social jetlag (SJL) in home-office compared to onsite-working students/employees.

## Methods

### Setting

This study was approved by the Eberhard Karls University’s ethics committee (Faculty of Economics and Social Sciences: no. A.Z.: A2.5.4-157_ns).

### Data collection

Data were collected from December 14, 2020, to January 18, 2022. Participants were recruited through university mailing lists and social media postings (Facebook, Instagram, WhatsApp). The study included a questionnaire that was available online on the SoSciSurvey platform (https://www.soscisurvey.de/). This hosting platform complies with European Union data protection regulations. Potential study participants were informed about the voluntary nature of participation, the possibility to stop data collection at any time without consequences, and the study compensation of 25 € in cash. Informed consent was obtained before data collection began. Exclusion criteria were shiftwork, participants who planned to travel across time zones during data collection, and participants who were not fluent in German.

### Variables and instruments

Participants reported information on their demographics (such as the work environment (“working onsite,” hereafter: “onsite group”; and “working from home,” hereafter: “home-office group”), age, sex, number of children in their household, occupation), details of their sleep behavior, and a morningness–eveningness questionnaire (the Morningness–Eveningness Stability Scale improved [MESSi]).

### MESSi

The MESSi consists of three subscales: morning affect (MA), eveningness (EV), and distinctness (DI). MA is designed to measure the affective component of morning orientation, while EV measures the affective component of evening orientation. In comparison to the diurnal changes measured by the MA and EV subscales, the DI subscale measures fluctuating changes in the expression of MA and EV [23].

### Actigraphy

Participants were asked to wear an ActiGraph (GT3X+, ActiGraph, Pensacola, FL, USA) for 7 consecutive days in order to measure sleep–wake patterns. The device is worn on the nondominant wrist similar to a wristwatch. At the end of the measurement week, participants returned their ActiGraphs and the data were downloaded using the accompanying software (ActiLife). Activity counts were collected in 1‑min epochs over a continuous period of 1 week (7 × 24 h). Any recordings that did not meet the minimum requirements of a) at least 10 h of valid signal during waking hours and b) a total of at least 4 valid days were excluded. Sleep–wake patterns were scored manually using the ActiLife software as described by Mitchell and colleagues [24]. Briefly, sleep onset and offset times were identified by diary report and observation of a sharp decrease/increase in activity. If there were discrepancies of more than 30 min between the sleep diary report and the decrease/increase in activity counts measured by the ActiGraph, sleep phases were determined visually based on the activity changes. We used the Cole–Kripke algorithm to estimate sleep and wake periods [25]. Non-wearing times were determined using the Troiano algorithm and compared with the sleep diary [26].

### Sleep diary

Participants completed a sleep diary during the week of actigraphic monitoring. Participants were asked to note bedtimes, naps during the day, awakenings during the night (duration and frequency), and times when the ActiGraph was taken off (e.g., to take a shower).

### Midpoint of sleep as a measure of chronotype

As a second measure of chronotype the corrected midpoint of sleep (MSFsc) as proposed by Roenneberg and colleagues was used [27]. This chronotype measure is based on clock time but adjusts for average individual sleep need [28]. It was shown to be a stable measure of chronotype during the COVID-19 pandemic in a similar German sample from Baden-Württemberg [13]. Throughout this manuscript the abbreviation “MPOS” represents “midpoint of sleep,” while in the following formula, “SDu” stands for “average sleep duration”:

$$\textit{MSFsc}=\mathrm{MPOS}_{\text{weekend}}-0.5\times (SDu_{\,\text{weekend}}-(5\times SDu_{\text{workdays}}+2\times SDu_{\text{weekend}})\div 7)$$

### Social jetlag

SJL is a parameter to describe the discrepancy between a person’s social and biological clocks due to circadian preferences [29]. Weekend sleep patterns are thought to reflect the internal biological clock, whereas weekday sleep patterns reflect the social clock. SJL is calculated by subtracting the midpoint of sleep on workdays from the midpoint of sleep on days off:

$$SJL=\mathrm{MPOS}_{\text{weekend}}-M\mathrm{POS}_{\text{workdays}}$$

### Sleep efficiency

Sleep efficiency is calculated by dividing the amount of time spent asleep (in minutes) by the total amount of time in bed (in minutes).

### Sleep time variability

The variability in sleep times was compared between the groups. This was done by calculating the standard deviation of sleep times for each participant and using this to calculate a group mean. The result shows the average standard deviation of sleep times and is considered the variability of sleep timing in the group.

### Statistical analyses

We further analyzed the actigraphy data using pyActigraphy [30], an open-source Python actigraphy analysis and visualization framework. In addition, averages of various sleep parameters were calculated for each subject using the Python data analysis library Pandas [31]. To calculate time averages after midnight, e.g., sleep onset time and midpoint of sleep, we added 24 h to times below an appropriate pivot element before calculating the mean, and then subtracted 24 h afterwards, if necessary. The software and a detailed description are available as open source at https://github.com/spuetz/actigraphy.git. Statistical analysis of the actigraphy and questionnaire data was performed using SPSS 29.0 software (IBM Corp., Armonk, NY, USA). Independent-sample *t*-tests and paired-sample tests were calculated to compare inter-and intra-individual differences. Furthermore, a multivariate general model was calculated with sex and work environment as fixed factors and age as a covariate to control the reliability of the *t*-tests. Kolmogorov–Smirnov and Shapiro–Wilk tests were used to test the normal distribution of the MSFsc variable. Levine tests indicated that equal variances could be assumed. Pearson’s chi-squared test was used for descriptive group comparisons.

## Results

Descriptive group statistics are presented in Table 1. Exploratory data analysis was performed to identify outliers in the age variable, as this variable has a strong influence on other variables. Outlier limits were defined as ±3 standard deviations from the mean value. Two cases were excluded in each group. This reduced the original sample size from *N* = 79 to *N* = 75. Overall, the home-office group was significantly younger than the onsite group. Significantly more participants were students in the home-office group compared to the onsite group. We did not see any difference between groups regarding children in the household or chronotype. The sleep timing variability did not differ between the groups. In terms of intra-individual differences in the variability of sleep times, the onsite group had the lowest standard deviation of 0.17 h for bedtimes and also the highest of 2.32 h. For rise times, the minimum and maximum were 0.2 h and 2.07 h (Table 1). In the home-office group, the minimum for bedtime was 0.2 h and the maximum was 1.9 h, while the rise times were 0.25 h and 2.1 h respectively (see Table 1).

**Table 1:** Demographics: onsite versus home-office group

Variable		Overall (*N* = 75)	Onsite group (*N* = 40)	Home-office group (*N* = 35)
Age, years, mean (SD)		28.2 (7.7)	30.3 (8.7)*	25.8 (5.6)*
Sex, *N* (%)	Male	24 (32.0)	15 (37.5)	9 (25.7)
	Female	51 (68.0)	25 (62.5)	26 (74.3)
Children in household, *N* (%)	Yes	5 (6.7)	4 (10.0)	1 (2.9)
	No	70 (93.3)	36 (90.0)	34 (97.1)
Profession, *N* (%)	Student	32 (42.7)	9 (22.5)*	23 (65.7)*
	Employee	37 (49.3)	25 (62.5)	12 (34.3)
	Not specified	6 (8.0)	6 (15.0)	–
Morningness, mean (SD)		3.37 (0.79)	3.41 (0.77)	3.32 (0.82)
Eveningness, mean (SD)		3.01 (0.86)	2.97 (0.83)	3.07 (0.90)
Distinctness, mean (SD)		3.31 (0.71)	3.35 (0.71)	3.27 (0.72)
Sleep time variability—bedtimes, mean (SD), max/min [h]		0.87 (0.45), 0.17/2.32	0.92 (0.45), 0.17/2.32	0.83 (0.43), 0.2/2.07
Sleep time variability—rise times, mean (SD), max/min [h]		0.95 (0.43), 0.2/2.1	0.93 (0.45), 0.2/1.9	0.95 (0.43), 0.25/2.1

*SD* standard deviation

*Significant differences between onsite and home-office (*p* < 0.05)

Comparisons of sleep patterns are shown in Table 2. No differences were seen in total time in bed, sleep efficiency, and total sleep time on weekdays, weekends, and all days. The home-office group went to bed, reached the midpoint of sleep, and got up significantly later than the onsite group on weekdays. Interestingly, sleep parameters did not differ between groups at weekends. Also, the variability of sleep timing (standard deviations of sleep timing, Table 1) did not differ between the groups. Both groups showed a SJL of about 1 h, which was not significantly different. In addition, there was no difference in chronotype measured by the MESSi scales MA/EV/DI (Table 1) or MSFsc (Table 2) between the two groups. For both the onsite and home-office groups, the Kolmogorov–Smirnov test and the Shapiro–Wilk test indicated a normal distribution of the MSFsc.

**Table 2:** Comparison of onsite versus home-office groups regarding sleep patterns

Characteristic	Onsite group (*N* = 40)			Home-office group (*N* = 35)			*p*-value
	Mean	SD	Std. error mean	Mean	SD	Std. error mean	
Average total time in bed (all days), h	7.65	0.78	0.2	7.73	0.77	0.2	0.636
Average total time in bed (workdays), h	7.55	0.92	0.22	7.63	0.87	0.22	0.685
Average total time in bed (weekend), h	7.90	1.12	0.28	8.00	1.02	0.28	0.695
Average total sleep time (all days), h	6.92	0.8	0.12	6.93	0.68	0.1	0.899
Average total sleep time (workdays), h	6.83	0.83	0.12	6.82	0.77	0.12	0.980
Average total sleep time (weekend), h	7.15	1.28	0.2	7.23	0.88	0.15	0.723
Average sleep efficiency (all days), %	90.4	0.05	0.01	89.9	0.05	0.01	0.605
Average efficiency (workdays), %	90.5	0.04	0.01	89.5	0.05	0.01	0.331
Average efficiency (weekend), %	90.1	0.09	0.01	90.7	0.05	0.01	0.738
Average bedtime (all days)	23:43	0.97	0.15	00:16	1.08	0.18	0.023*
Average bedtime (workdays)	23:28	0.98	0.15	00:07	1.05	0.17	0.008*
Average bedtime (weekend)	00:21	1.3	0.2	00:45	1.33	0.22	0.197
Average midpoint of sleep all days	3:14	0.92	0.13	3:48	1.0	0.17	0.015*
Average midpoint of sleep workdays	2:57	0.97	0.15	3:33	0.97	0.15	0.009*
Average midpoint of sleep weekend	3:59	1.17	0.18	4:25	1.18	0.2	0.122
Average corrected midpoint of sleep	3:51	1.27	0.2	4:17	1.28	0.22	0.158
Average rise time (all days)	7:25	1.0	0.15	8:01	1.05	0.17	0.014*
Average rise time (workdays)	7:05	1.18	0.18	7:44	1.13	0.18	0.019*
Average rise time (weekend)	8:17	1.12	0.17	8:47	1.2	0.2	0.068
Social Jetlag, h	1.03	1.02	0.15	0.87	0.72	0.12	0.403

*SD* standard deviation

*Significant differences between onsite and home-office (*p* < 0.05)

As age differed significantly between the onsite and home-office groups (30.3 versus 25.8 years, *p* = 0.01), a multivariate general linear model adjusting for age was applied (fixed factors: sex and work environment) (Table 3).

**Table 3:** Results of the general linear model controlling for age as a covariate

Dependent variable		F	*p*-value	Estimated marginal means	
				Mean	Std. error
Average total time in bed (all days), h	Onsite	0.006	0.938	7.65	0.13
	Home-office			7.63	0.13
Average total time in bed (workdays), h	Onsite	0.068	0.795	7.57	0.15
	Home-office			7.52	0.17
Average total time in bed (weekend), h	Onsite	0.147	0.702	7.83	0.18
	Home-office			7.93	0.2
Average total sleep time (all days), h	Onsite	0.258	0.613	6.88	0.12
	Home-office			6.80	0.13
Average total sleep time (workdays), h	Onsite	0.575	0.451	6.82	0.13
	Home-office			6.67	0.15
Average total sleep time (weekend), h	Onsite	0.042	0.837	7.05	0.18
	Home-office			7.12	0.20
Average efficiency (all days), %	Onsite	0.511	0.477	0.90	0.008
	Home-office			0.89	0.008
Average efficiency (workdays), %	Onsite	1.183	0.280	0.90	0.007
	Home-office			0.89	0.008
Average efficiency (weekend), %	Onsite	0.006	0.940	0.90	0.012
	Home-office			0.90	0.013
Average bedtime (all days)	Onsite	5.034	0.028*	23:51	00:10
	Home-office			00:23	00:11
Average bedtime (workdays)	Onsite	7.225	0.009*	23:37	00:10
	Home-office			00:15	00:11
Average bedtime (weekend)	Onsite	1.615	0.208	00:26	00:13
	Home-office			00:50	00:14
Average midpoint of sleep all days	Onsite	4.522	0.037*	03:21	00:09
	Home-office			03:50	00:10
Average midpoint of sleep workdays	Onsite	5.022	0.028*	03:04	00:09
	Home-office			03:35	00:10
Average midpoint of sleep weekend	Onsite	2.103	0.151	04:01	00:11
	Home-office			04:27	00:13
Average corrected midpoint of sleep	Onsite	1.362	0.247	03:55	00:13
	Home-office			04:17	00:14
Average rise time (all days)	Onsite	4.338	0.041*	07:32	00:10
	Home-office			08:03	00:11
Average rise time (workdays)	Onsite	3.528	0.064	07:14	00:11
	Home-office			07:45	00:12
Average rise time (weekend)	Onsite	3.173	0.079	08:18	00:12
	Home-office			08:49	00:13
Social jetlag, h	Onsite	0.179	0.673	0.95	0.15
	Home-office			0.85	0.17

Means of squares are given as a decimal number

*Significant differences between onsite and home-office

In comparison with the independent-sample test (Table 2), the general linear model confirmed the significant findings. The only exception was the variable “average rise time workdays,” which was no longer significant in the general linear model.

Sleep parameters on weekdays were also compared with weekend days to investigate intra-individual differences within each group using paired-sample *t*-tests (Table 4). Intra-individual differences were found in both the onsite and home-office groups. The sleep parameters of the onsite group differed significantly between weekdays and weekend days with regard to sleep efficiency, bedtime, total sleep time, rise time, and midpoint of sleep. The home-office group differed in the same sleep parameters except for total sleep time.

**Table 4:** Intra-individual differences between sleep parameters on weekdays versus weekends

Characteristic	Onsite group (*N* = 40)			Home-office group (*N* = 35)		
	Mean	SD	*p*-value	Mean	SD	*p*-value
Average total time in bed	−0.33	1.30	0.208	−0.35	1.10	0.058
Average total sleep time	−0.32	1.28	0.037*	−0.40	0.97	0.063
Average efficiency	0.004	0.07	< 0.001*	−0.01	0.04	< 0.001*
Average bedtime	−0.87	1.03	< 0.001*	−0.63	0.75	< 0.001*
Average midpoint of sleep	−1.03	1.02	< 0.001*	−0.87	0.72	< 0.001*
Average rise time	−1.20	1.30	0.026*	−1.05	1.05	< 0.001*

*SD* standard deviation

*Significant differences between weekdays and weekend days (*p* < 0.05)

Onsite workers had a higher sleep efficiency on weekdays compared to weekends, while home-office workers had a higher sleep efficiency on weekends compared to weekdays. Average bedtime and midpoint of sleep were significantly later at weekends compared to weekdays for both groups.

## Discussion

In summary, it was found that onsite workers had significantly earlier sleep timing and midpoints of sleep on weekdays compared to home-office workers, whereas there were no clinically meaningful differences regarding sleep efficiency, sleep duration, and variability in sleep timing. Importantly, also SJL did not differ between the groups. The results of the group comparison showed that the onsite group was significantly older than the home-office group. The group means differed by 4.5 years. This age difference is particularly important when considering the present results. Chronotype, as an individual difference trait, is subject to changes in expression over the course of a person’s life [32]. While young children tend to be morning types, this tendency changes strongly during adolescence [32]. Adolescents show a distinct delay in chronotype, peaking at the age of 20 years [27]. With the end of adolescence, the daytime preference shifts again and begins to advance. Roenneberg and colleagues [27] showed a continuous advancement of chronotype from the age of 20 until about 65 years. There is also a steeper regression line between the ages of 20 and 45 years than between 45 and 65 years. Most adults do not belong to an extreme chronotype and are therefore described as neither type [32]. In the sample of Randler and colleagues [32], a distribution of 21.1% evening, 71.7% neither, and 7.2% morning type was reported for the 26-year-old subjects. Among the 30-year-old subjects, 11.9% were classified as evening type, 74.8% as neither, and 13.3% as morning type. This means that the 30-year-old subjects were 9.2% less likely to be classified as evening type than the 26-year-old subjects. As the average age of the present sample groups differed by about 4.5 years (mean 25.8 years in home-office, mean 30.3 years in onsite), it could be assumed that also the chronotypes differ significantly from each other. Participants in the home-office group may therefore be more evening oriented, reflecting a higher proportion of evening types in this group than in the onsite group.

In this study two different measures of chronotype were used (MSFsc: clock time-based measure, MESSi: continuous morningness-eveningness measure). The analysis of the MSFsc and the MESSi showed no significant differences between the groups. Therefore, the expression of chronotype did not differ between the groups.

Regarding the MSFsc variable, Roenneberg and colleagues [33] described a comparable shape and width of the distribution in all age groups, with a varying mean value. Graphical comparison of the group histograms (see Supplement 1) with the expected values ([33], *N* ≥ 55,000) showed that the distribution in the home-office group had a similar shape. In the onsite group, two equally high peaks existed, one at 3:00 a.m. and one at 4:30 a.m., showing the shape of the distribution to be different from the reference. In the sample of Roenneberg and colleagues [33], the 26-year-old subjects showed peaks at around 4:45 a.m. compared to 4:17 a.m. for the home-office group. For the 30-year-old participants, Roenneberg and colleagues [33] reported an average MSFsc of approximately 4:30 a.m., compared to 3:51 a.m. for the onsite group. Taken together, these two discrepancies led us to consider the MSFsc of the onsite group to be unrepresentative.

In summary, a) the expected distribution of chronotype based on the age groups of the samples and b) the finding that the distribution of chronotype in the onsite group cannot be considered representative suggest that the chronotypes of the groups can generally be expected to differ. The younger group would normally have a higher prevalence of evening types/more pronounced eveningness, although this was not the case in the present samples.

Increased eveningness is associated with a higher risk of SJL. SJL has repeatedly been shown to be particularly detrimental to public health (e.g., cardiovascular outcomes, psychiatric disorders, obesity [34]). However, softer factors such as productivity at work are also significantly affected by SJL. To test whether the measured SJL in the home-office group was within the expected range, the results were compared to an earlier sample of the authors’ research group [13]. In that study, only people who worked in home-office during the COVID-19 pandemic had been surveyed. The mean age of the sample was reported to be ~ 28.6 years (SD: ~ 10.5 years), which was not different compared to the sample of the present study (*p* = 0.11; Supplement 2). With regard to SJL, the means of the present home-office group (0.80, SD: 0.73 h) and the previous sample (0.82; SD: 0.70 h) were also not significantly different (*p* = 0.87; Supplement 2). Furthermore, the chronotype within the two groups measured by MSFsc did not differ (*p* = 0.21; Supplement 2). It can therefore be assumed that the SJL in the present sample is representative for this age group working from home. In another sample of the same age who were not working from home, the SJL was almost 1 h higher (1.78 h) compared to the present sample with 0.8 h [35]. It could be postulated that the implementation of home-office might potentially be beneficial for young people to reduce SJL. The onsite group had an average SJL of 1.03 h, which was also compared to other samples in the current literature. As no age-matching sample could be found in the literature, the present results were compared to slightly younger and older samples. Both showed a meaningful but equal age difference compared to the present sample. Interestingly, the SJL (1.4 ± 1.0, [36]; 1.33 ± 0.88 h, [37]) of the two studies did not significantly differ (*p* = 0.42, t = 0.78, df = 727, Standard Error of Difference (SED) = 0.09). Therefore, these results were used as representative reference values for SJL in the current sample. Even the value closer to the current study [37] differed significantly from the SJL reported herein (*p* = 0.04, t = 2.07, df = 637, SED = 0.15; Supplement 2). This led to the conclusion that the SJL of the onsite group cannot be considered representative, as it was significantly below the expected range.

Although the SJL of the present two study groups were not significantly different, there was a tendency towards lower SJL in the home-office group. This tendency was reinforced by an overrepresentation of low SJL in the onsite group, while the home-office group showed more similar values to age-comparable samples. Therefore, the current results suggest that working from home might help to reduce SJL.

The present study differs from the authors’ previous studies which examined only home-office participants and not two samples in different working environments. As spatial flexibility, in addition to temporal flexibility [14], may play a role in the increasing irregularity of students’ sleep schedules, a possible relationship was investigated here.

The sleep timing of the two groups on workdays differed significantly. The home-office group went to bed later and also got up later. However, both groups had similar fluctuations regarding sleep timing variability and no difference in overall nightly sleep duration. Thus, home-office had no negative influence on sleep variability and duration in this sample. There was also no influence of spatial flexibility/working environment on the loss of sleep duration/regularity observed. Instead, data support the hypothesis that the ability to work with unrestricted temporal flexibility is the main influencing factor of the increased sleep timing regularity in students, as discussed in one of the authors’ previous studies [14] by excluding another possible influencing factor.

Sleep efficiency did not differ between the two groups. However, a difference within the groups could be detected. In the onsite group, sleep efficiency was higher on weekdays than at weekends. The opposite was true for the home-office group, where sleep efficiency was higher at weekends. The reason for this could be explained by the way the sleep efficiency variable is calculated. On weekdays, participants in the onsite group could have either gotten up immediately after waking up (e.g., because of the increased pressure to get up on time in order to commute to work compared to the weekend) or gone straight to sleep after going to bed (e.g., because of the increased exhaustion from onsite work with probably fewer breaks compared to the weekend). This would explain the high ratio of total sleep time to time in bed, which corresponds to a higher sleep efficiency. At the weekend, on the other hand, they may have stayed in bed even longer although they were already awake, or it took longer for them to fall asleep, resulting in lower sleep efficiency. In contrast, the opposite was true for the home-office group, with a higher sleep efficiency at weekends compared to weekdays. Although statistically significant, this effect was small and not clinically meaningful.

## Conclusion

In summary, it was found that home-office workers had a delay in sleep timing that did not have a clinically meaningful effect on any other sleep parameter such as sleep efficiency or nightly sleep duration. Compared to values in the current literature, SJL seemed to be reduced in the home-office group. Overall, the work environment had only marginal impact on sleep patterns and thus sleep health in this sample. Sleep timing variability did not differ between groups.

## Limitations and strengths

Strengths of the study were the objective data collection via actigraphy and the assessment of different sleep variables covering sleep quantity, quality, and circadian patterns. The results of the study were limited by the fact that the sample was relatively small and there was a significant age difference between the groups. In addition, students were overrepresented in the home-office group. Moreover, although the data were collected during the same seasons, which reduces seasonal effects, they were collected over a long period of time, this may have influenced the data in terms of the evolution of the pandemic (different severity of lockdown regimes, psychological effects due to different familiarity and understanding).

## Supplementary Information

Supplement 1 (Fig. 1: Distribution of the corrected midpoint of sleep as a chronotype measure in the onsite group; Fig. 2: Distribution of the corrected midpoint of sleep as a chronotype measure in the home-office group), Supplement 2 (Table: Comparison of age and social jetlag with literature)

## Declarations

For this article no studies with animals were performed by any of the authors. All studies mentioned were in accordance with the ethical standards indicated in each case.
