# Supplementary material for: Onsite versus home-office: differences in sleep patterns according to workplace
Source: Somnologie (Berl). 2023 Jun 6:1–8. Online ahead of print. doi: 10.1007/s11818-023-00408-5 (PMC10243697; doi:10.1007/s11818-023-00408-5)
Supplement: Supplementary file 1 — Supplement 1 (Fig. 1: Distribution of the corrected midpoint of sleep as a chronotype measure in the onsite group; Fig. 2: Distribution of the corrected midpoint of sleep as a chronotype measure in the home-office group), Supplement 2 (Table: Comparison of age and social jetlag with literature) [file 11818_2023_408_MOESM1_ESM.docx]

***ESM***

# **Supplement 1**

For both the onsite and home-office groups, the Kolmogorov-Smirnov test and the Shapiro-Wilk test indicate a normal distribution of the MSFsc but the graphical distribution as shown in figures 1 and 2 differ from each other.

Figure 1: Distribution of the corrected Midpoint of Sleep as a chronotype measure in the onsite group.

Figure 2: Distribution of the corrected Midpoint of Sleep as a chronotype measure in the home-office group.

# **Supplement 2**

| Characteristic | Mean | SD | N | Mean | SD | N | p-value |
| --- | --- | --- | --- | --- | --- | --- | --- |
|  | Present study: home-office group | | | Literature: [13] | | |  |
| Age | 25.8 | 5.6 | 35 | ~28.6 | ~10.5 | 681 | 0.11 |
| Social Jetlag | 0.8 | 0.73 |  | 0.82 | 0.7 |  | 0.87 |
| MSFsc | 4.28 | 1.28 |  | 4.6 | 1.48 |  | 0.21 |
|  | Literature: [36] | | | Literature: [37] | | |  |
| Age | 33.6 | 8.8 | 130 | 28.16 | 4.4 | 599 | **<0.001** |
| Social Jetlag | 1.04 | 1.01 |  | 1.33 | 0.88 |  | 0.42 |
|  | Present study: onsite group | | | Literature: [37] | | |  |
| Age | 30.3 | 8.7 | 40 | 28.16 | 4.40 | 599 | **0.01** |
| Social Jetlag | 1.03 | 1.02 |  | 1.33 | 0.88 |  | **0.04** |
